# An intensive, structured, mobile devices-based healthcare intervention to optimize the lipid-lowering therapy improves lipid control after an acute coronary syndrome

**DOI:** 10.3389/fcvm.2022.916031

**Published:** 2022-07-26

**Authors:** Sonia Ruiz-Bustillo, Neus Badosa, Ignacio Cabrera-Aguilera, Consol Ivern, Marc Llagostera, Diana Mojón, Miren Vicente, Núria Ribas, Lluis Recasens, Julio Martí-Almor, Mercè Cladellas, Núria Farré

**Affiliations:** ^1^Heart Diseases Biomedical Research Group, IMIM (Hospital del Mar Medical Research Institute), Barcelona, Spain; ^2^Cardiac Rehabilitation Unit, Department of Cardiology, Hospital del Mar, Barcelona, Spain; ^3^Departament of Medicine, Autonomous University of Barcelona, Barcelona, Spain; ^4^Department of Medicine, Pompeu Fabra University, Barcelona, Spain; ^5^Unitat de Biofísica i Bioenginyeria, Facultat de Medicina i Ciéncies de la Salut, Universitat de Barcelona, Barcelona, Spain; ^6^Department of Human Movement Sciences, Faculty of Health Sciences, School of Kinesiology, Universidad de Talca, Talca, Chile

**Keywords:** ischemic heart disease, secondary prevention, cardiovascular risk factors, lipid-lowering therapy, mobile devices-based healthcare

## Abstract

**Aims:**

Despite the evidence, lipid-lowering treatment (LLT) in secondary prevention remains insufficient, and a low percentage of patients achieve the recommended LDL cholesterol (LDLc) levels by the guidelines. We aimed to evaluate the efficacy of an intensive, mobile devices-based healthcare lipid-lowering intervention after hospital discharge in patients hospitalized for acute coronary syndrome (ACS).

**Methods and results:**

Ambiespective register in which a mobile devices-based healthcare intervention including periodic follow-up, serial lipid level controls, and optimization of lipid-lowering therapy, if appropriate, was assessed in terms of serum lipid-level control at 12 weeks after discharge. A total of 497 patients, of which 462 (93%) correctly adhered to the optimization protocol, were included in the analysis. At the end of the optimization period, 327 (70.7%) patients had LDLc levels ≤ 70 mg/dL. 40% of patients in the LDLc ≤ 70 mg/dL group were upgraded to very-high intensity lipid-lowering ability therapy vs. 60.7% in the LDLc > 70 mg/dL group, *p* < 0.001. Overall, 38.5% of patients had at least a change in their LLT. Side effects were relatively infrequent (10.7%). At 1-year follow-up, LDLc levels were measured by the primary care physician in 342 (68.8%) of the whole cohort of 497 patients. In this group, 71.1% of patients had LDLc levels ≤ 70 mg/dL.

**Conclusion:**

An intensive, structured, mobile devices-based healthcare intervention after an ACS is associated with more than 70% of patients reaching the LDLc levels recommended by the clinical guidelines. In patients with LDLc measured at 1-year follow-up, 71.1% had LDLc levels ≤ 70 mg/dL.

## Introduction

There is extensive evidence confirming the benefit of cardiac rehabilitation programs (CRP) in patients with ischemic heart disease (IHD) (1). An intensive CRP reduces cardiovascular risk through lifestyle changes (2). However, correct pharmacological treatment is also crucial for optimal cardiovascular risk factors control, including dyslipidemia. The drop in low-density lipoprotein cholesterol (LDLc) levels in secondary prevention significantly reduces mortality, coronary events, coronary revascularization procedures, and ischemic strokes (3).

Despite the evidence, lipid-lowering treatment (LLT) in secondary prevention remains insufficient, and a low percentage of patients achieve the LDLc levels recommended by the guidelines (4–6). One factor explaining this contradiction is the so-called therapeutic inertia, defined as the failure of physicians to initiate or intensify an indicated therapy (7). Other significant barriers are lack of infrastructure and availability of CRP, lack of perceived importance of secondary prevention among professionals, and low patient motivation and financial difficulties to pay the LLT (8).

Mobile devices-based healthcare (mHealth) aimed at improving patients’ living standards (9) can be an effective tool to improve the suboptimal results in dyslipidemia control (10). Considering the disappointing results in secondary prevention, we conducted a randomized pilot study to evaluate whether an intensive mHealth lipid-lowering intervention implemented after a hospitalization due to IHD was associated with a lower LDLc level. We demonstrated that this strategy was associated with improved management of LDLc levels compared with standard care alone (11). Our current study aimed to assess whether this same structured mHealth-based protocol helped improve LDLc levels after a hospitalization due to IHD in a real-life population.

## Materials and methods

### Study design and participants

The Risk Optimization Acute Coronary Syndrome (RiskOp-ACS) study was a single-center ambispective register assessing the efficacy and safety of a lipid-lowering intervention to improve the management of LDLc levels in patients hospitalized for IHD (ClinicalTrials.gov Identifier: NCT03619395). Between July 2018 and September 2019, all patients hospitalized for IHD in our hospital not meeting any exclusion criteria (inability or refusal to sign the informed consent or presenting comorbidities with a life expectancy of less than 1 year) and willing to participate in the CRP were screened for inclusion in this study. All patients providing written informed consent were included in the RiskOp-ACS register. The Ethics Committee of the Hospital del Mar approved the study and was conducted per the Declaration of Helsinki. The Ethics Committee approved the retrospective inclusion of patients who followed the same protocol since November 2016 to increase the sample size and waived the need for written informed consent. Therefore, all patients included in the study (both prospectively and retrospectively) followed the same optimization protocol.

### Cardiac rehabilitation program

The multidisciplinary CRP performed in our center is coordinated by specialized nurses. It includes interventions performed by cardiologists, nurses, rehabilitation physicians, and professionals specialized in managing anxiety and other mental health disorders. All patients discharged after a hospitalization for an acute IHD event and with no severe cognitive impairment are invited to the CRP. As part of the program activities, nurses educate patients in healthy habits during the in-hospital stage and at follow-up visits at 3 and 12 months after discharge; provide and monitor the quality of life, anxiety, and depression symptoms using validated tests, and coordinate the follow-up plan and visits. Rehabilitation physicians and physiotherapists assess the patient’s functional status and indicate and supervise physical activity during follow-up. All professionals involved in the CRP participate in monthly group sessions aimed at reinforcing the health education of the patients, with a particular focus on increasing the patients’ understanding of the pathophysiology of IHD, on the role of cardiovascular risk factors, and the importance of optimal risk factor management, mainly through physical activity, control of anxiety, and adherence to guideline-recommended pharmacotherapies.

### Intervention

All patients were discharged under LLT. Serum lipid levels were measured at week 6 after discharge, and a virtual visit with the cardiologist was made within 1 week of the blood test. The laboratory test results were evaluated using a pre-specified algorithm based on clinical practice guidelines (Supplementary Figure 1). If there was a need to modify the LLT, the electronic prescription was changed accordingly, and the patient was informed by phone. Thus, the protocol avoided the need to come to the hospital or primary care center since the medication could be retrieved directly from the local pharmacy or printed/seen in the personal health record through a dedicated app/website created by the Health Department (*Lamevasalut*). This intervention was repeated every 6 weeks after every pharmacological change until the target LDLc levels (calculated by the Friedewald formula) advised in the clinical guidelines were achieved, or maximum lipid-lowering therapy according to local regulation was reached. Thus, the duration of the intervention was a maximum of 3 months (“optimization period”). From that moment, the follow-up was performed by the patient’s primary care general physician and primary care cardiologist. Creatinine kinase and liver tests were measured in all blood tests following LLT modification.

The LLT intensity was defined according to its ability to reduce LDLc (12, 13). The moderate lipid-lowering ability group comprises moderate-intensity statins and low-intensity statin plus ezetimibe. High-intensity statins, and medium-intensity statins plus ezetimibe are the high-reduction group. High-potency statins plus ezetimibe are the very-high reduction group. Finally, PCSK9 inhibitor added at maximally tolerated doses to LLT is considered extreme reduction ability treatment (Supplementary Table 1). In our country, the prescription of PCSK9 inhibitors is allowed in the public health system for patients with established cardiovascular disease and no optimal control defined as LDLc > 100 mg/dL despite the maximum tolerated dose of statins, patients intolerant to statins, or in whom statins are contraindicated (14).

### Study endpoints

The primary study endpoint was the proportion of patients with serum LDLc levels ≤ 70 mg/dL at the end of the optimization period, which was the treatment goal supported by current ESC guidelines (15) when the study was conducted. Other variables related to lipid management assessed during the intervention included lipid-lowering medication use, changes in lipid-lowering medication, and the presence of side effects, among others. As an exploratory analysis, the number of patients who reached LDLc levels < 55 mg/dL at the end of the optimization period was also analyzed. Finally, we assessed also as an exploratory analysis, clinical outcomes with the achievement of LDL ≤ 70 mg/dL at the end of the optimization period.

### Statistical analyses

Data for continuous variables are expressed as mean ± standard deviation (SD) or median and interquartile range (IQR) based on normality distribution assessed by Kolmogorov-Smirnov test. Categorical variables were expressed as percentages. Differences in baseline characteristics between groups were tested using the χ^2^-test (categorical variables) and Student’s *t*-test or Mann–Whitney *U*-test, one-way analysis of variance, or Kruskal-Wallis test for continuous variables. All analysis was performed using IBM SPSS Statistics v25 (Armonk, NY, United States). For all tests, *p* < 0.05 was considered as statically significant.

## Results

The whole cohort included 497 patients, of which 462 (93%) correctly adhered to the optimization protocol and were included in the analysis. At the end of the optimization period, 327 (70.7%) patients had LDLc levels ≤ 70 mg/dL, and 159 (34.7%) had LDLc levels < 55 mg/dL.

According to LDLc levels achieved, baseline characteristics of patients are described in Table 1. Interestingly, the only differences between both groups were a higher prevalence of chronic obstructive pulmonary disease in the LDLc ≤ 70 mg/dL group and different history of smoking.

**TABLE 1:** Baseline characteristics of patients according to LDLc levels after the optimization protocol.

	LDLc ≤ 70 mg/dL (*n* = 327)	LDLc > 70 mg/dL (*n* = 135)	*P*-value
Age (years)	62.9 ± 11.6	62.9 ± 11.9	0.98
Women (*n*, %)	56 (17.1)	30 (22.9)	0.15
BMI (Kg/m2)	27.7 ± 4.0	27.7 ± 4.6	0.95
**Risk factors and comorbidities**			
Hypertension (*n*, %)	185 (56.6)	72 (55)	0.75
Hyperlipidemia (*n*, %)	207 (63.3)	94 (71.8)	0.09
Diabetes mellitus (*n*, %)	100 (30.6)	33 (25.2)	0.25
Current smoker (*n*, %)	119 (36.4)	65 (49.6)	0.008
Previous smoker > 1 year (*n*, %)	114 (34.9)	29 (22.1)	
Previous smoker < 1 year (*n*, %)	16 (4.9)	2 (1.5)	
Previous ACS-MI (*n*, %)	60 (18.3)	25 (19.1)	0.86
COPD (*n*, %)	32 (9.8)	5 (3.8)	0.02
Cerebrovascular disease (*n*, %)	18 (5.5)	5 (3.8)	0.46
Peripheral vascular disease (*n*, %)	25 (7.6)	9 (6.9)	0.78
Anemia (*n*, %)	53 (16.2)	28 (21.4)	0.19
Chronic kidney disease (*n*, %)	21 (6.4)	7 (5.3)	0.66
**Index hospitalization**			
STEMI (*n*, %)	140 (42.8)	60 (45.8)	0.79
NSTEMI (*n*, %)	118 (36.1)	43 (32.8)	
Unstable Angina (*n*, %)	69 (21.1)	28 (21.4)	
One vessel disease (*n*, %)	170 (52)	76 (58)	0.29
Two vessel disease (*n*, %)	85 (26)	28 (21.4)	
Three vessel disease (*n*, %)	63 (19.3)	20 (15.3)	
Left main disease (*n*, %)	14 (4.3)	9 (6.9)	0.25
Coronary percutaneous angioplasty (*n*, %)	280 (85.6)	107 (81.7)	0.29
Ejection fraction (%)	56.4 ± 10.0	55.6 ± 10.2	0.42

Data are mean ± SD, median (IQR), or n (%). BMI, Body mass index; COPD, chronic obstructive pulmonary disease; CV, Cardiovascular; STEMI, ST-elevation myocardial infarction; NSTEMI, non-ST-elevation myocardial infarction; ACS-MI, Acute coronary syndrome-myocardial infarction.

Table 2 shows LDLc levels at baseline and during follow-up, the type of LLT given, and the presence of side effects. Interestingly, LLT did not differ at discharge from the hospital, with 65% of patients on high lipid-lowering ability treatment. However, by the end of the optimization therapy, 40% of patients in the LDL ≤ 70 mg/dL levels were upgraded to very-high intensity lipid-lowering ability vs. 60.7% in the LDL levels > 70 mg/dL, *p* < 0.001. Overall, 38.5% of patients had at least a change in their LLT. The LDLc levels > 70 mg/dL group had a higher number of changes in medications (61.4 vs. 27.5%, *p* < 0.001). However, this treatment modification was not enough to achieve good LDLc control in this group. During the optimization period, only 14 (2.8%) received extreme lipid-lowering ability treatment with PCSK-9 inhibitors, 5 (1.5%) patients in the LDLc ≤ 70 mg/dL, and 9 (6.9%) patients in the LDL levels > 70 mg/dL, *p* = 0.003.

**TABLE 2:** Baseline LDL levels and medication changes according to LDL levels reached.

	LDLc ≤ 70 mg/dL (*n* = 327)	LDLc > 70 mg/dL (*n* = 135)	*P*-value
Baseline LDLc	103.9 ± 36.6	115.2 ± 45.2	0.012
End of optimization LDLc	53.4 ± 10.1	85.0 ± 16.7	< 0.001
Δ LDLc at the end of optimization (absolute reduction)	−50.0 (−21, −77)	−31.0 (+ 2, −53)	< 0.001
Δ LDLc at the end of optimization (relative reduction)	−47.5 (−25, −60.1)	−26.2 (+ 2.6, −41.0)	< 0.001
1-year LDLc levels	61.5 ± 18.3	69.9 ± 25.4	0.003
**Baseline LLT**			
Moderate lowering ability (*n*, %)	17 (5.3)	7 (5.4)	0.21
High lowering ability (*n*, %)	206 (63.8)	88 (68.2)	
Very high lowering ability (*n*, %)	96 (29.7)	30 (23.3)	
**LLT end of optimization**			
Moderate lowering ability (*n*, %)	15 (4.5)	3 (2.2)	< 0.001
High lowering ability (*n*, %)	171 (52.2)	40 (29.6)	
Very high lowering ability (*n*, %)	131 (40)	82 (60,7)	
Extreme lowering ability (*n*, %)	5 (1.5)	10 (7.4)	
Number of side effects reported (*n*, %)	38 (11.8)	15 (11.6)	0.97
**Side effects**			
Liver test abnormalities (*n*, %)	24 (7.3)	6 (4.4)	0.30
Creatine Kinase increase (*n*, %)	6 (1.8)	4 (2.9)	
Myalgia (*n*, %)	3 (0.9)	4 (2.9)	
Change in LLT (*n*, %)	90 (27.5)	83 (61.4)	< 0.001
**LLT at 1 year**			
Moderate lowering ability (*n*, %)	11 (4.5)	2 (2)	0.46
High lowering ability (*n*, %)	113 (46.1)	45 (45)	
Very high lowering ability (*n*, %)	117 (47.8)	51 (51)	

Data are mean ± SD, median (IQR), or n (%). LDLc, low-density lipoprotein cholesterol LLT; lipid lowering therapy.

Side effects were relatively infrequent, with 53 reports (10.7%) of the cohort (patients could have more than one side effect reported). The more common side effects were abnormal asymptomatic liver tests, increased creatine-kinase levels, and myalgia. However, these side effects were rarely associated with LLT discontinuation. In most cases, side effects led to a change or temporary suspension in LLT in 4.3% of patients, without differences in both groups.

At 1-year follow-up, LDLc levels were measured by the local cardiologist or primary care physician in 342 (68.8%) of the whole cohort of 497 patients. In this group, 71.1% of patients had LDLc levels ≤ 70 mg/dL. Interestingly, 77.4% of patients with LDL ≤ 70 mg/dL at the end of optimization still had LDL levels ≤ 70 mg/dL at 1-year follow-up, whereas 59.6% of patients who did not reach LDLc ≤ 70 mg/dL did so at 1 year.

At a median follow-up of 30 (p25–75: 21–38) months, patients who achieved an LDLc ≤ 70 mg/dL after the optimization protocol had a numerically lower incidence of myocardial infarction [12 (3.6%) patients vs. 10 (7.5%), *p* = 0.08], need of new revascularization [22 (6.7%) patients vs. 10 (7.5%), *p* = 0.77), death (12 (3.6%) patients vs. 8 (6%), *p* = 0.27], and the composite end-point that comprised the 3 outcomes [33 (10%) vs. 22 (16.4%), *p* = 0.054] compared to those who did not achieve an LDLc ≤ 70 mg/dL.

## Discussion

Our study showed that an intensive, structured, mHealth post-discharge follow-up plan to optimize the lipid-lowering pharmacotherapy was associated with achieving a target LDLc ≤ 70 mg/dL in 70.7% of patients after an acute coronary syndrome (ACS). Reduction and achievement of LDLc target value were obtained early (between 6 and 12 weeks after the ACS) as recommended in the literature (16). It is also important to emphasize that the intervention was carried out using low-cost phone-based telemedicine techniques (mHealth) that facilitate its implementation and patient follow-up. The use of mHealth might explain why less than 8% of the patients did not adhere to follow-up.

Considering the few significant baseline characteristics differences between the LDLc ≤ 70 mg/dL and LDLc > 70 mg/dL groups, these differences did not allow us to identify those patients who would achieve proper LDLc control. It is worth mentioning that the percentage of patients achieving LDLc ≤ 70 mg/dL in this real-life study is even better than the results we obtained in a previous randomized study in which we used the same strategy, where an LDLc ≤ 70 mg/dL was achieved in 62% of the patients (10). Moreover, these results contrast positively with the poor results described in our hospital before implementing the study intervention (4). This insufficient control of dyslipidemia in very high-risk patients coincides with that described more recently in the literature. The ESC-EORP Euroaspire V survey performed in 27 European countries showed that the prevalence of LDLc ≤ 70 mg/dL in the entire cohort was 30% (5). Similar findings were found in a retrospective analysis of patients with ACS in Finland, where two-thirds of patients on statin therapy did not achieve the LDLc level target recommended by the guidelines (6). According to these data, our strategy could more than double the number of patients with adequate LDLc level control after a hospitalization due to IHD.

The most recent ESC/EAS guidelines for the management of dyslipidemias published in 2019 advise an LDLc < 55 mg/dL for high and very high-risk patients. The recommendations regarding the treatment goals for LDLc are based in the studies that have shown that the lower the LDLc level the better (17). These guidelines were not published during the performance of our study. In our cohort, 34.7% of the patients achieved an LDLc < 55 mg/dL. It is worth noting that it was not the target level of our study and, thus, there was some room for LLT optimization. This result improves that reported in the DA VINCI study; being one of the first comparative analyses evaluating 2019 risk-based goal attainment, showed that three-quarters of patients did not meet their 2019 LDLc goals (18).

The inability to achieve an LDLc level ≤ 70 mg/dl was not due to therapeutic inertia. Intensifications or treatment changes were done in 64% of the patients. When changes were not made, patients were already on the maximum dose of oral treatment, did not tolerate a higher dose or did not meet the local criteria for using PCSK9 inhibitors. This intensification effort is superior to the ones described in the literature. The GOULD prospective observational registry study was carried out simultaneously in the United States. A total of 5,006 patients with established atherosclerotic cardiovascular disease were enrolled. Surprisingly, only 17% had LLT intensification after 2 years, while two-thirds remained at an LDLc level > 70 mg/dL (19). Another important finding in our study is that 95% of the patients at the end of the optimization period were treated with drugs included in the high, very high, or extreme lipid-lowering ability groups. In the register published by Navar et al., of almost 3,297 patients analyzed, only 47% of the patients who required secondary prevention were treated with the appropriate intensity of treatment (20). One of the hypotheses that may justify not achieving an optimal decrease in LDLc, despite treatment is the described lack of response to statin treatment. A meta-analysis of genome-wide association studies showed that two loci of the genome are responsible for about 5% of the variation in an individual’s response to statin treatment (21). The JUPITER study highlights that 43% of high-risk patients had an LDLc reduction < 50 and 11% showed no reduction or even an increase in LDLc with statin treatment (22).

Large cardiovascular outcomes trials showed a prognostic benefit with PCSK9 inhibitors monoclonal antibodies (23, 24), and it has been estimated that 30% of patients could be candidates of PCSK9 inhibitors (25). However, the use of PCSK9 inhibitors in our study was low due to prescription restrictions in our country. PCSK9 inhibition obtains further lowering of LDLc beyond that achieved with statin therapy and cholesterol absorption inhibitors; an incremental reduction in LDLc of 50% from baseline has been shown (26). Knowing that the LDLc levels in the group with LDLc > 70 mg/dL were 85.0 ± 16.7 mg/dL and the hypothetical additional reduction of 50%, we theorized that we could have even achieved 100% of patients with LDLc target levels if we had been allowed to expand the use of PCSK9 inhibitors.

Numerous studies with lipid-lowering drugs have shown that the reduction of LDLc levels translates into a reduction in cardiovascular events, giving rise to the current recommendations in secondary prevention (17). We saw that patients who achieved an LDLc ≤ 70 mg/dL after the optimization protocol had numerically fewer clinical endpoints. However, it is important to remark that the study was underpowered to detect hard endpoint due to the relatively small sample size and the number of events.

One of the reasons that might limit LLT is side effects. Some physicians and patients might be reluctant to begin or maintain statins, most commonly because of perceived side effects that are not confirmed (i.e., the nocebo effect) (27). However, statins are generally well-tolerated drugs (25). 11% of the patients had any side effects in our cohort, with no significant differences between the LDLc group ≤ 70 mg/dL and the LDLc group > 70 mg/dL. The most frequent side effect was the liver tests abnormalities (79%), followed by an increase in creatinine kinase (26%) and finally myalgias (18%). These data are comparable to those previously described (15, 28).

After the treatment optimization period, the patient’s primary care general physician and primary care cardiologist performed the follow-up. We decided to analyze whether the good results achieved in the first 3 months were maintained after 12 months. Surprisingly, 31% of the patients did not have a follow-up blood test at month 12. In those patients who did have an LDLc assessment at 1 year, 71.1% of the patients maintained an LDLc ≤ 70 mg/dl. This group comprises 77.4% of the patients who already had an LDLc ≤ 70 mg/dl initially and patients who had not reached this target level originally (59.6%). This percentage of patients with a target LDLc level after 12 months remains significantly higher than the results reported in the literature (4–6).

Our study used the simplest and most readily usable form of mHealth, such as telephone calls. We believe that it was a determining factor in the success of this follow-up protocol. A randomized study in Sweden (29) and a meta-analysis of randomized controlled trials (10) showed improved LDLc levels with mHealth.

### Limitations

As a single-center study, the results might not apply to other settings. Some of the patients were included retrospectively, potentially leading to bias. Still, given that all patients followed the same protocol and that the information was documented in the medical record, we believe that the risk of bias in this study is negligible.

## Conclusion

An intensive, structured, mobile devices-based healthcare intervention after an ACS is associated with more than 70% of patients reaching the LDLc levels recommended by the clinical guidelines. This strategy uses low-cost and easy-to-apply telemedicine techniques that can be replicated in various clinical settings.

## Data availability statement

The original contributions presented in this study are included in the article/Supplementary material, further inquiries can be directed to the corresponding author.

## Ethics statement

The patients/participants prospectively included in the study provided their written informed consent. The patients/participants included retrospectively did not require informed consent after evaluation by the Research Ethics Committee of the Hospital del Mar.

## Author contributions

SR-B: conceptualization and writing original draft preparation. SR-B and NF: methodology, project administration, and validation. NF: formal analysis and writing – review and editing. SR-B, NB, CI, NF, and NR: investigation. IC-A, MV, DM, ML, LR, SR-B, and NF: data curation. NB, CI, IC-A, DM, MV, ML, NR, LR, JM-A, and MC: visualization. NF, JM-A, and MC: supervision. All authors contributed to the article and approved the submitted version.

## Supplementary material

SUPPLEMENTARY FIGURE 1 Lipid-lowering treatment optimization algorithm.
