# Supplementary material for: An intensive, structured, mobile devices-based healthcare intervention to optimize the lipid-lowering therapy improves lipid control after an acute coronary syndrome
Source: Front Cardiovasc Med. 2022 Jul 26;9:916031. doi: 10.3389/fcvm.2022.916031 (PMC9360604; doi:10.3389/fcvm.2022.916031)
Supplement: Supplementary file 1 [file Table_1.DOCX]

**Supplementary Material**

**Table S1.** Classification of lipid-lowering drugs according to their ability to reduce low-density lipoprotein cholesterol [13]

| **Lowering LDL ability** | **Treatment** |
| --- | --- |
| Extreme reduction | PCSK9 inhibitor added to maximally tolerated doses of lipid-lowering treatment  Evolocumab 140 mg  Alirocumab 75 mg  Alirocumab 150 mg |
| Very high reduction | High-potency statin + ezetimibe  Atorvastatin 40-80 mg + ezetimibe 10 mg Rosuvastatin 10-40 mg + ezetimibe 10 mg |
| High reduction | High-potency statin  Atorvastatin 40-80 mg  Rosuvastatin 20-40 mg  Medium-potency statin + ezetimibe  Simvastatin 20-40 mg + ezetimibe 10 mg  Pravastatin 40 mg + ezetimibe 10 mg  Lovastatin 40 mg + ezetimibe 10 mg  Fluvastatin 80mg + ezetimibe 10mg  Pitavastatin 2-4 mg + ezetimibe 10 mg  Atorvastatin 10-20 mg + ezetimibe 10 mg  Rosuvastatin 5mg + ezetimibe 10mg |
| Moderate reduction | Medium-potency statin  Atorvastatin 10-20 mg  Rosuvastatin 5-10 mg  Simvastatin 20-40 mg  Pravastatin 40 mg  Lovastatin 40 mg  Pitavastatin 2-4 mg  Fluvastatin XL 80 mg  Low-potency statin + ezetimibe  Simvastatin 10mg + ezetimibe 10 mg  Pravastatin 20 mg + ezetimibe 10 mg  Lovastatin 20 mg + ezetimibe 10 mg  Fluvastatin 40mg + ezetimibe 10mg  Pitavastatin 1 mg + ezetimibe 10mg |
